# Engineering Cell Microenvironment Using Nanopattern-Derived Multicellular Spheroids and Photo-Crosslinked Gelatin/Hyaluronan Hydrogels

**DOI:** 10.3390/polym15081925

**Published:** 2023-04-18

**Authors:** Zhen Zhang, Yi Liu, Xuelian Tao, Ping Du, Myagmartsend Enkhbat, Khoon S. Lim, Huaiyu Wang, Peng-Yuan Wang

**Affiliations:** 1 Shenzhen Key Laboratory of Biomimetic Materials and Cellular Immunomodulation, Shenzhen Institute of Advanced Technology, Chinese Academy of Sciences, Shenzhen 518055, China; 2 University of Chinese Academy of Sciences, Beijing 100049, China; 3 Shenzhen Key Laboratory of Smart Healthcare Engineering, Department of Biomedical Engineering, Southern University of Science and Technology, Shenzhen 518055, China; 4 School of Medical Sciences, University of Sydney, Sydney, NSW 2052, Australia; 5 Oujiang Laboratory, Key Laboratory of Alzheimer’s Disease of Zhejiang Province, Institute of Aging, Wenzhou Medical University, Wenzhou 325000, China

**Keywords:** nanopattern, cell spheroids, HBMSC, HUVEC, hydrogel

## Abstract

Cell cultures of dispersed cells within hydrogels depict the interaction of the cell–extracellular matrix (ECM) in 3D, while the coculture of different cells within spheroids combines both the effects of cell–cell and cell–ECM interactions. In this study, the cell co-spheroids of human bone mesenchymal stem cells/human umbilical vein endothelial cells (HBMSCs/HUVECs) are prepared with the assistance of a nanopattern, named colloidal self-assembled patterns (cSAPs), which is superior to low-adhesion surfaces. A phenol-modified gelatin/hyaluronan (Gel-Ph/HA-Ph) hydrogel is used to encapsulate the multicellular spheroids and the constructs are photo-crosslinked using blue light. The results show that Gel-Ph/HA-Ph hydrogels with a 5%-to-0.3% ratio have the best properties. Cells in HBMSC/HUVEC co-spheroids are more favorable for osteogenic differentiation (Runx2, ALP, Col1a1 and OPN) and vascular network formation (CD31+ cells) compared to HBMSC spheroids. In a subcutaneous nude mouse model, the HBMSC/HUVEC co-spheroids showed better performance than HBMSC spheroids in angiogenesis and the development of blood vessels. Overall, this study paves a new way for using nanopatterns, cell coculturing and hydrogel technology for the generation and application of multicellular spheroids.

## 1. Introduction

Human tissues are a variety of cells within a 3D microenvironment. For example, bone is a highly vascularized tissue, including mesenchymal stromal cells (MSCs), osteoblast precursor cells, macrophages, pericytes and endothelial progenitor cells (EPCs) [1]. For the in vitro biomimetic study of bone tissues, two or more bone-related cells should be cocultured within a scaffold. However, the traditional coculture method requires the dispersal of a variety of cells in the scaffold, which cannot simulate the in vivo microenvironment due to the lack of direct cell–cell contact. Therefore, finding a suitable scaffold is the key to studying cell–cell and cell–extracellular matrix (ECM) connections.

Hydrogels, which provide several advantages, such as a high-water content, uniform 3D network structure and good biocompatibility [2], are quite conforming to the characteristics of the ECM. Encapsulating cells in hydrogels is a facile way to maintain the 3D microenvironment of implants for tissue repair. In addition, hydrogels using a mixture of proteins and polysaccharides have been successfully utilized as delivery systems for drugs and bioactive ingredients, standing out for their favorable biological characteristics and tunable physical properties [3,4]. There are numerous other examples of biomedical applications of hydrogels, which are not otherwise enumerated.

Thanks to the emergence of organoid technologies, multicellular spheroids assembled with the use of different types of cells have been developed for research in artificial tissue construction [5,6,7], tumor therapy [8,9,10] and tissue repair [11,12,13]. Cells can produce specific ECM and paracrine factors within the cell spheroids depending on the culture conditions. The coculture of different cells within cell spheroids can provide sufficient cell–cell interactions, which may be beneficial to cell differentiation and tissue development. Compared with the methodologies of 2D cultures, 3D cultures within cell spheroids can promote self-renewal, differentiation activity and upregulate the paracrine of MSCs [14]. More growth factors, such as FGF1, VEGF and PGE2, can be secreted within spheroids, which can be favorable to angiogenesis and wound healing, and alleviate the immune response [15,16]. Therefore, multicellular spheroids have shown good promise as a novel cell coculture system in studying cell–cell connections.

The current technologies for fabricating cell spheroids include hanging drops, spinner culture and ultra-low-adhesion surfaces. These methods are based on the principle that cells cannot attach to substrates and then attach to each other to survive [17,18], as a result suffering from disadvantages such as a low efficiency, nonuniform sizes and time-consuming protocols. More importantly, to our knowledge, forcing cells to attach to each other may induce some negative effects, for example, multicellular spheroids prepared through external force can cause mechanical damage to cells [11]. On the other hand, if the cells can form spheroids on a nanopatterned substrate, the external forces on cells can be alleviated [19,20,21]. In our previous studies, it was demonstrated that colloidal self-assembled patterns (cSAPs) are versatile cell culture substrates capable of regulating the propagation, proliferation, polarization and differentiation of various cells [22,23,24]. The cSAPs can even be used to modulate the adhesion and migration of tumor cells and MSCs, resulting in the spontaneous formation of multicellular spheroids [25,26]. The remarkable advantages of such a strategy are the high efficiency of spheroidization and the convenience of spheroid collection. Based on this, we developed a novel nanopatterned substrate for the preparation of multicellular spheroids, with which we expect to prepare HBMSC/HUVEC co-spheroids to study their biological activity in vitro and in vivo, and to highlight the cell–cell connections in cocultures.

**Scheme 1:**
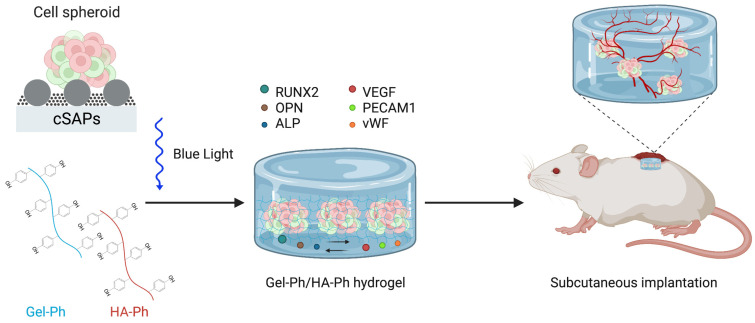
A graphic outline of this study. Cell spheroids were formed through coculturing human bone mesenchymal stem cells (HBMSCs) and human umbilical vein endothelial cells (HUVECs) on a type of nanopattern, cSAPs. Cells were attached to the cSAPs and migrated to form cell spheroids. Then, cell spheroids were encapsulated within a mixture of phenol-functionalized gelatin (Gel-Ph) and hyaluronan (HA-Ph) hydrogels photo-crosslinked using a blue light. Osteogenesis and angiogenesis were characterized in the osteogenic or angiogenic/osteogenic induction medium for 7 and 14 days. Finally, angiogenesis of the cell spheroids (4 weeks) was characterized in a subcutaneous nude mouse model.

Here, in this study, we aimed to generate an optimal 3D cell microenvironment for tissue generation, such as osteogenesis and angiogenesis. At the same time, we aimed to understand whether cell–cell interactions could improve their functions (Scheme 1). First, cell spheroids were formed through coculturing human bone mesenchymal stem cells (HBMSCs) and human umbilical vein endothelial cells (HUVECs) on a type of nanopattern, called cSAPs. Cells were attached to the cSAPs and migrated to form cell spheroids that are more biomimic than that on a noncell adhesion surface, in our opinion. Then, cell spheroids were encapsulated within a mixture of phenol-functionalized gelatin (Gel-Ph) and hyaluronan (HA-Ph) hydrogels photo-crosslinked using a blue light. Osteogenesis and angiogenesis were characterized in the osteogenic or angiogenic/osteogenic induction medium for 7 and 14 days. Finally, the angiogenesis of the cell spheroids was characterized in a subcutaneous nude mouse model for 4 weeks. Our study paves a way for studying cell–cell interactions, facilitating organoid research and tissue engineering.

## 2. Materials and Methods

### 2.1. Materials

SiO_2_ and polystyrene (PS) particles were purchased from Bangs Laboratories, Inc. Gelatin, hyaluronan, 3-(4-hydroxyphenyl) propionic acid, N-(3-dimethylaminopropyl)-N′-ethylcarbodiimide hydrochloride (EDC), N-hydroxysuccinimide (NHS), tris(2,2′-bipyridyl) dichlororuthenium (II) hexahydrate [Ru(bpy)_3_Cl_2_] and sodium persulphate (Na_2_S_2_O_8_) were all obtained from Sigma-Aldrich (St. Louis, MO, USA). The gelatin (pig skin, type A) and sodium hyaluronic acid (HA, average molecular weight, approximately 1880 kDa) were purchased from Yeasen (Shanghai, China) and Kewpie (Tokyo, Japan), respectively.

### 2.2. Synthesis of Gelatin-Phenol (Gel-Ph)

The synthesis of Gel-Ph was modified according to a previous study [27,28]. In total, 1 g of gelatin was dissolved in 20 mL of MES buffer (pH 4.5, 25 mM), and 3-(4-hydroxyphenyl) propionic acid (50 mg), N-(3-dimethylaminopropyl)-N′-ethylcarbodiimide hydrochloride (EDC; 58 mg) and N-hydroxysuccinimide (NHS; 35 mg) were dissolved in 80 mL of MES buffer. Two solutions were mixed to react at 37 °C in the dark for 24 h. The resulting solution was purified using dialysis against purified water, and the final product was lyophilized and stored at −20 °C.

### 2.3. Synthesis of Hyaluronan-Phenol (HA-Ph)

The synthesis of HA-Ph was modified according to a previous study [27,29]. In total, 100 mg of hyaluronan was dissolved in 40 mL of MES buffer. EDC (50 mg) and NHS (30 mg) were added to the hyaluronan solution; then, a tyramine solution (18 mg in 10 mL of MES buffer) was added to the mixture. The reaction proceeded for 24 h in the dark while stirring at room temperature. The synthesized product was dialyzed against purified water, and the final product was isolated through freeze-drying.

### 2.4. Characterization of Gel-Ph and HA-Ph

A proton nuclear magnetic resonance (^1^H NMR) analysis and UV–Vis measurement were performed to identify the Gel-Ph and HA-Ph. A total of 10 mg/mL of Gel-Ph and HA-Ph solutions in D_2_O was measured using an NMR spectrometer (AVIII-500 MHz FT-NMR, Bruker, MA, USA). In total, 1 mg/mL of Gel-Ph and HA-Ph solutions in DI water was scanned at wavelengths from 200 nm to 600 nm using a UV–Vis spectrometer (SpectraMax M5, Molecular Devices, San Jose, CA, USA). The absorbance peak at 275 nm was ascribed to the phenol group.

### 2.5. Preparation of Gel-Ph/HA-Ph Hydrogels

Gel-Ph and HA-Ph were dissolved in phosphate-buffered saline (PBS, pH 7.4). The Gel-Ph/HA-Ph hydrogels were prepared by mixing 5% (wt%) of a Gel-Ph solution with a HA-Ph solution at different concentrations (0%, 0.1%, 0.3% and 0.5%, wt%) at a volume ratio of 1:1.

To prepare the photo-crosslinkable Gel-Ph/HA-Ph hydrogels, 0.1 mM of tris(2,2′-bipyridyl) dichlororuthenium (II) hexahydrate [Ru(bpy)_3_Cl_2_] and 3 mM of sodium persulphate (Na_2_S_2_O_8_) were added into the Gel-Ph/HA-Ph solutions, respectively. For photo-crosslinking, the hydrogel solution was radiated for 1 min under a blue-light-emitting diode (LED,440~460 nm, 7 W, Vitalux, Taiwan) from a distance of ~5 cm.

### 2.6. Characterization of Gel-Ph/HA-Ph Hydrogels

#### 2.6.1. SEM

The concentration of Gel-Ph was fixed at 5% (wt%) and the concentration of HA-Ph was gradually increased (0%, 0.1%, 0.3% and 0.5%, wt%) to prepare four kinds of Gel-Ph/HA-Ph hydrogels, which were named GH_0_, GH_1_, GH_3_ and GH_5_, respectively. The obtained hydrogels were snap frozen in liquid nitrogen, dried in a freeze-dryer (CHRIST SZM-41, Osterode, Germany) for 24 h and then examined for internal morphologies with FE-SEM (Carl Zeiss Supra 55, Jena, Germany).

#### 2.6.2. Compression Test

The compression test was performed at room temperature using the micromechanical test system (IBTC-300SL, CARE Measurement & Control, Tianjin, China). Each hydrogel sample was prepared in a cylindrical shape (8 mm diameter and 3.5 mm height) and compressed at a rate of 4 mm/min until ~95% strain. Young’s modulus was calculated from the slope in the linear region from 0 to 10% strain. Crushing stress was determined from the first drop of the stress value.

#### 2.6.3. Rheological Analysis

The viscoelasticity of the hydrogel was measured using a rheometer (MCR 302, Anton Paar) with a 20 mm parallel-plate geometry. The hydrogel was freshly prepared and added onto the Peltier plate immediately when the environmental temperature was controlled to 25 °C. For the time-dependent analysis, the storage moduli (G′) and loss moduli (G″) of the hydrogels were measured at a 0.1 Hz frequency and 1% dynamic strain. To investigate the effect of visible light on the crosslinking, the rheological properties of the hydrogels upon light exposure were measured in situ with the time sweep experiment using a light-guide accessory and a mercury lamp (320~500 nm, ~50 mW/cm^2^, Omnicure S2000, Exfo, Montreal, QC, Canada) at a 0.1 Hz frequency and 1% dynamic strain. The light irradiation was applied for 100 s after 120 s of gelling time.

#### 2.6.4. Swelling

Each kind of hydrogel was prepared as mentioned above and 150 μL of the pre-hydrogel solution in PBS was placed in a 2 mL centrifuge tube. Subsequently, the obtained hydrogels were incubated in 1.5 mL of PBS at 37 °C. The swelling property was determined by examining the wet weight of each hydrogel after a certain number of days. The percentage of wet weight was calculated using the equation (Ws/Wi) × 100%, where Wi and Ws represent the wet weight of each hydrogel at day 0 and the wet weight of the swollen hydrogel at a certain timepoint, respectively.

### 2.7. Cell Culture

The HBMSCs were purchased from Cyagen Biosciences (HUXMF-01,001, Suzhou Inc., Suzhou, China) at passage 2. Cells were routinely cultured in an α-MEM supplemented with 10% fetal bovine serum (FBS, Gibco, NSW, Australia) and 1% penicillin/streptomycin. The HUVECs were cultured in Vascular Cell Basal Medium (ATCC^®^ PCS-100-030) supplemented with an Endothelial Cell Growth Kit-VEGF (ATCC^®^ PCS-100-041). Cells were cultured at 37 °C in a humidified incubator with 5% CO_2_. Upon 80% confluency, cells were detached using trypsin/EDTA (0.25% *w*/*v* trypsin/0.02% EDTA, Gibco) for cell seeding.

### 2.8. Cell Viability in Gel-Ph/HA-Ph Hydrogels

The HBMSCs were suspended in different compositions of Gel-Ph/HA-Ph solutions (GH_0_, GH_1_, GH_3_, GH_5_) containing 0.1 mM [Ru(bpy)_3_Cl_2_] and 3 mM Na_2_S_2_O_8_ at a cell density of 3.0 × 10^4^ cells/mL. The cells suspended in the polymer were dropped into a 24-well plate and crosslinked via blue light irradiation for 1 min. Afterwards, the cell culture medium was added, and cell proliferation was detected using a CCK-8 assay after cultivation for 1, 4, 7 and 14 days. In addition, cells were stained with 2 μM Calcein-AM and 4.5 μM propidium iodide (PI) to evaluate their viability on day 5.

### 2.9. Formation of Multicellular Spheroids Using cSAPs

cSAPs were fabricated through capillarity-assisted particle assembly in a confined area. In particular, 24-well tissue culture plates were treated with air plasma (0.4 mbar, 200 W and 3 min); then, 100 μL of mixing solutions with SiO_2_ and PS particles were drop-casted in the substrate of a 24-well tissue culture plate. The samples were transferred to a confined area and left at room temperature to dry. The obtained cSAPs were stabilized through heating at 95 °C for 2 h, and then fixed via dipping in a toluene solution and quickly being washed with ethanol before further use.

The HBMSCs with or without HUVECs were inoculated in a 24-well plate coated with cSAP particles at a density of 1 × 10^5^ cells/mL (cell numbers in HBMSCs/HUVECs were equivalent), respectively. The culture plates were then slowly placed in an incubator allowing the cells cultured on the cSAPs to automatically assemble into multicellular spheroids within 48 h.

### 2.10. Live/Dead Assay

Upon the formation of cell spheroids on the cSAPs, the medium was carefully removed and 2 μM Calcein-AM and 4.5 μM PI were added for 30 min to evaluate cell viability. The stained samples were observed and imaged under a fluorescent microscope (Olympus, Tokyo, Japan) for simultaneously detecting the live cells (yellow–green fluorescence) and dead cells (red fluorescence) in spheroids.

### 2.11. Dynamic Monitoring of Multicellular Spheroids’ Self-Assembly Process on cSAPs

The process of the self-assembly formation of multicellular spheroids was monitored using the JuLI Stage Living cell monitoring system fitted with an incubator. Prior to photography, HBMSC and HUVEC cells were stained with the cell membrane dyes PKH67 and PKH26 at a concentration of 1 × 10^−6^ M, respectively, and the stained cells were seeded on cSAPs at a density of 1 × 10^5^ cells/mL. After the cells were allowed to adhere for 1 h, the camera took pictures at 1 h intervals for 48 h.

### 2.12. Encapsulation of Multicellular Spheroids in the Gel-Ph/HA-Ph Hydrogels

The multicellular spheroids were harvested from cSAPs and mixed with Gel-Ph/HA-Ph (formula of GH_3_) solutions containing 0.1 mM [Ru(bpy)_3_Cl_2_] and 3 mM Na_2_S_2_O_8_. Afterwards, the mixtures were dropped into a 24-well plate and crosslinked with blue light irradiation for 1 min. For the angiogenic/osteogenic induction, the samples were cultured for two weeks in an osteogenic medium (α-MEM supplemented with 10% fetal bovine serum, 1% PS, 50 μg/mL L-ascorbic acid, 10 mM β-glycerophosphate and 100 nM dexamethasone) mixed with Vascular Cell Growth Medium at a ratio of 1:1. The medium was changed every other day and the growth status of the multicellular spheroids was photographed at day 0 and day 7 under a microscope.

### 2.13. Angiogenic/Osteogenic Differentiation In Vitro

#### 2.13.1. Immunofluorescence Staining

For the immunofluorescent staining, the Gel-Ph/HA-Ph hydrogels incorporated with HBMSC spheroids and HBMSC/HUVEC co-spheroids were fixed in 4% paraformaldehyde (PFA) for 20 min at room temperature, permeabilized with 0.2% Triton X-100 for 15 min and blocked with 3% bovine serum albumin (BSA) for 1 h, followed by overnight incubation at 4 °C with antibodies specific for CD31 (1:100, Abcam, Cambridge, UK) or Col lα1 (1:400, Abcam). Subsequently, the appropriate secondary antibodies (diluted 1: 1000) were used, and cell nuclei were stained with 4,6-diamidino-2-phenylindole (DAPI, 1:1000, Invitrogen, Carlsbad, CA, USA) for 5 min. The stained samples were visualized using an LSM-710 laser-scanning confocal microscope (Zeiss, Jena, Germany).

#### 2.13.2. Gene Expression Assay

The Gel-Ph/HA-Ph hydrogels loaded with multicellular spheroids were prepared for in vitro cultures as mentioned above. The total mRNA was extracted from the multicellular spheroids using the TRIzol reagent (Invitrogen, USA). Afterwards, mRNA was subjected to cDNA synthesis using the 5× PrimeScript RT Master Mix (TaKaRa, RR036A) at 37 °C for 15 min and 85 °C for 5 s, according to the manufacturer’s protocol. The expression of target genes was quantified with a real-time quantitative polymerase chain reaction (RT-qPCR) using the SYBR Green master (Genestar, A311) with a LightCycler^®^96 Real-Time PCR System (Roche, Basel, Switzerland). The relative gene expression was calculated using the 2(−ΔΔCT) method when GAPDH served as the house-keeping gene. The following primers in Table 1 were utilized for the RT-qPCR.

### 2.14. In Vivo Evaluation

#### 2.14.1. Subcutaneous Implantation in Nude Mice

Male BALB/c mice (4–5 weeks old, 20 ± 2 g) were purchased from Zhuhai Best Test Bio-Tech Co., Ltd. (Zhuhai, China). For the in vivo experiments, we used a 1.5 mL EP tube lid (8 mm diameter and 3.5 mm height) as a mold to prepare hydrogels of equal volume in size with or without cell spheroids. A total of 18 nude mice were randomly divided into three groups, including the hydrogel group (control), the hydrogel group loaded with HBMSC spheroids (HBMSC) and the hydrogel group loaded with HBMSC/HUVEC co-spheroids (HBMSC/HUVEC). Firstly, a 1–1.5 cm wound was cut on the back skin of the nude mice, followed by the implantation of the as-prepared hydrogels under the skin on the back of the nude mice; finally, the wound was sutured. The body weight of the experimental mice was recorded and the volume of the hydrogels at the implantation site was measured every 4 days. In total, 9 nude mice were sacrificed after 2 and 4 weeks of implantation, respectively. The dorsal skin of the implantation site was carefully dissected, and the skin tissue and residual hydrogel were separated and immediately fixed in 10% formalin for histopathological identification.

#### 2.14.2. Histological Analysis

The samples fixed in 10% formalin were embedded in paraffin and cut at 5 μm intervals. The specimens were heated at 60 °C for 3 h, deparaffinized in xylene and rehydrated in ethanol series. After being rinsed with PBS 3 times, the tissue sections were stained with hematoxylin and eosin for 3 min, dehydrated and sealed with neutral gum. The stained sections were observed under a microscope (Olympus, Tokyo, Japan).

#### 2.14.3. Immunofluorescent Staining

For immunofluorescent staining, the paraffin-embedded tissue sections described above were deparaffinized in xylene and rehydrated in serial ethanol. The slides were permeabilized with 0.2% Triton X-100 for 15 min and blocked with 3% bovine serum albumin (BSA) for 1 h at 37 °C, followed by overnight incubation at 4 °C with antibodies specific for CD31 (1:100, Abcam, Cambridge, MA, USA). The appropriate secondary antibodies (diluted 1:1000) were then used, and the cell nuclei and F-actin were co-stained with 4,6-diamidino-2-phenylindole (DAPI, 1:1000, Invitrogen) and phalloidin (1:200, Invitrogen) for 5 min and 30 min, respectively. The stained samples were visualized using an LSM-710 laser-scanning confocal microscope (Zeiss, Jena, Germany).

### 2.15. Statistical Analysis

The quantitative data were presented as the mean ± standard deviation. Statistical analyses of the data were performed using an unpaired *t*-test and one-way ANOVA with Tukey’s post hoc multiple comparisons (GraphPad Prism 8). Statistical significance was expressed as * (*p* < 0.05), ** (*p* < 0.01) and *** (*p* < 0.001), respectively.

## 3. Results

### 3.1. Characterization of Gel-Ph and HA-Ph

**Figure 1:**
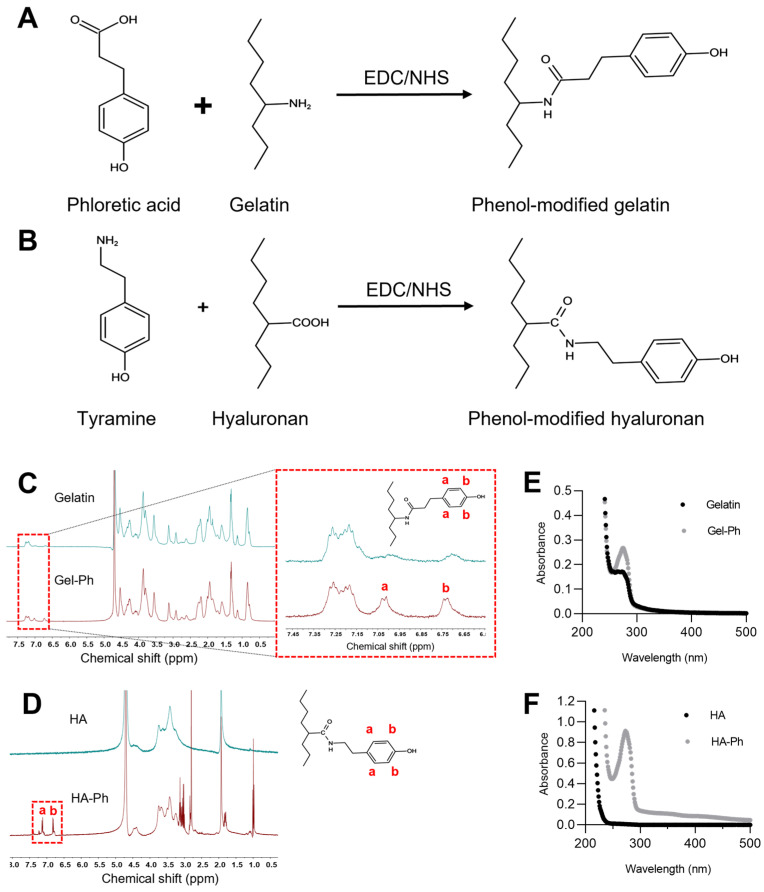
(**A**) Synthesis of Gel-Ph with EDC/NHS method; (**B**) synthesis of HA-Ph with EDC/NHS method; (**C**) ^1^H NMR spectrum of Gel-Ph; (**D**) ^1^H NMR spectrum of HA-Ph; (**E**) UV–Vis spectra of Gel-Ph solution; (**F**) UV–Vis spectra of HA-Ph solution.

According to previous studies, Gel-Ph and HA-Ph were synthesized via the conjugation of phloretic acid with gelatin and the conjugation of tyramine with hyaluronan (Figure 1A,B), respectively [27,29]. The ^1^H NMR spectrum of Gel-Ph and HA-Ph showed two peaks corresponding to phenol groups at ~6.8 and ~7.1 ppm, confirming the effective conjugation of phloretic acid and tyramine to the polymer backbones (Figure 1C,D) [27,28].The UV–Vis spectra analysis of Gel-Ph and HA-Ph displayed a significant absorbance peak at 275 nm, while such a peak was not detected for gelatin and hyaluronan (Figure 1E,F). The absorbance peak at 275 nm was attributed to the existence of phenol groups of Gel-Ph and HA-Ph [27,30,31,32,33,34]. These results indicated the successful introduction of phenol groups onto the gelatin and HA.

### 3.2. Characterization of Gel-Ph/HA-Ph Hydrogels

**Figure 2:**
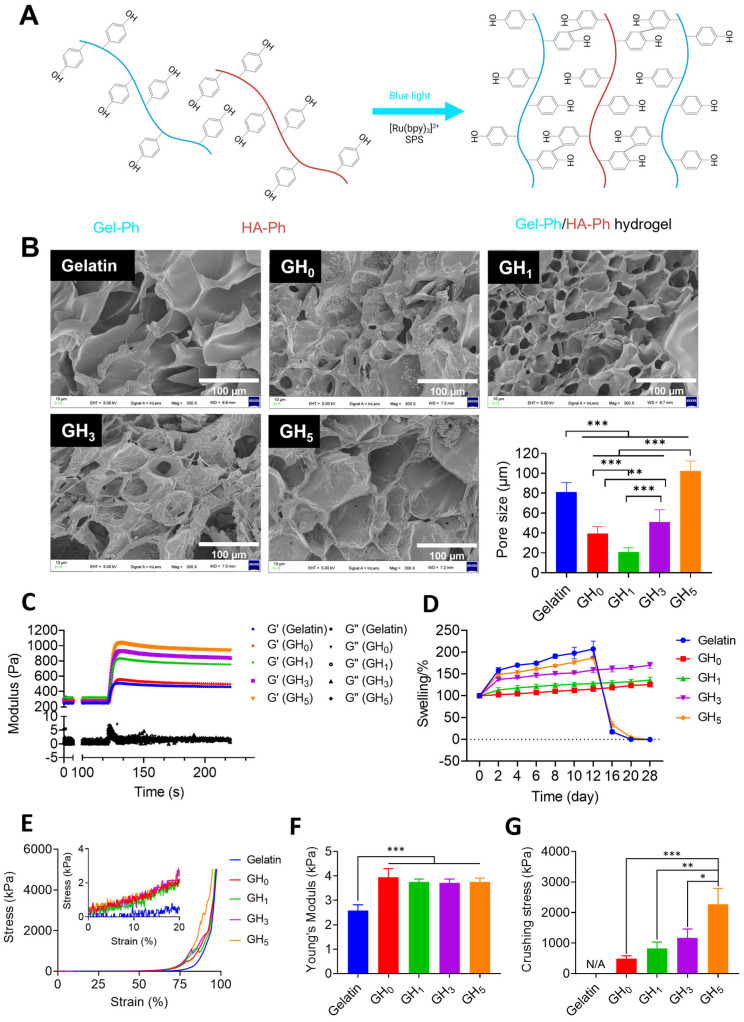
(**A**) Schematic representation of the formation of Gel-Ph/HA-Ph hydrogels; (**B**) SEM images of the internal pore structures and pore sizes of Gel-Ph/HA-Ph hydrogels; (**C**) rheological characteristics of Gel-Ph/HA-Ph hydrogels; (**D**) swelling characteristics of Gel-Ph/HA-Ph hydrogels; (**D**–**F**) compression test of Gel-Ph/HA-Ph hydrogels: (**D**) stress–strain curves, (**E**) Young’s moduli and (**F**) crushing stress values. (**G**) The crushing stress of the hydrogels represented the greatest compressive stress before the failure of the network (stress dropped). *, ** and *** represent *p* < 0.05, *p* < 0.01 and *p* < 0.001, respectively (*n* = 3).

The Gel-Ph/HA-Ph hydrogels were fabricated with varied contents of HA-Ph (GH_0_, GH_1_, GH_3_ and GH_5_) through the blue-light-initiated crosslinking of Ph moieties mediated with Ru and SPS (Figure 2A) [31,35]. The SEM images showed that the pore sizes of the Gel-Ph/HA-Ph hydrogels increased with the increase in the HA content, except that of GH_0_ and gelatin (Figure 2B), which was consistent with the previous report [36]. The averaged pore sizes of gelatin (81.0 ± 9.7 um), GH_0_ (39.3 ± 6.9 um), GH_1_ (20.7 ± 4.4 um), GH_3_ (50.8 ± 12.5 um) and GH_5_ (102.2 ± 10.2 um) showed that the phenol modification and low HA concentration (0.1%) decreased the pore size, while the pore size increased at high HA concentrations (>0.5%) due to the strong water absorption of HA.

The gelation dynamics of Gel-Ph/HA-Ph were characterized using rheology after crosslinking. The storage shear modulus G′ and loss shear modulus G″ of the Gel-Ph/HA-Ph hydrogels against gelling time are shown together in Figure 2C. It was clear that the G″ of Gel-Ph/HA-Ph with different components did not show any differences, which were all below 10 Pa. The crosslinking reaction started after 120 s of illumination, and the storage shear modulus (G′) of the Gel-Ph/HA-Ph hydrogels increased gradually and reached a peak. The stabilization time of the hydrogels in each group was essentially the same, which was contrary to the previous report [36]. The difference might be ascribed to the different crosslinking methods. Most importantly, the storage shear modulus of the Gel-Ph/HA-Ph hydrogels increased with the HA content, which was GH_5_ > GH_3_ > GH_1_ > GH_0_ > gelatin.

The influence of the hydrogel compositions on the swelling ratio of the hydrogels was also investigated. As shown in Figure 2D, five kinds of hydrogels with different components were immersed in PBS and tested for a period of 28 days. After 2 days of incubation, the wet weight of the hydrogels increased significantly in the gelatin, GH_3_ and GH_5_ groups, while it did not increase significantly in the GH_0_ and GH_1_ groups. It was observed that gelatin without modification and GH_5_ could swell significantly, with the swelling ratio reaching up to ~200% and ~190%, respectively. Even on day 12, the structure of the gelatin-only and GH_5_ hydrogel was destroyed due to water absorption and expansion, and, hence, the measurement could not be continued. Interestingly, the swelling ratio of the hydrogels in the other groups maintained a slow and steady growth trend over time. It was noteworthy that the swelling ratio increased from ~120% of GH_0_ to ~190% of GH_5_, which indicated that the swelling ratio of the hydrogels was positively correlated with the HA content.

The mechanical properties of the various hydrogels were characterized using a compression test. As shown in Figure 2E, the compressive stress of the hydrogels increased with compressive strain, and the Gel-Ph-based hydrogels showed a faster increase in stress compared to the gelatin hydrogel. Moreover, the Gel-Ph/HA-Ph hydrogels with a higher HA-Ph content needed more stress to deform, particularly at a certain high level of strain. The compressive Young’s moduli of the hydrogels were calculated from their initial linear regions. As shown in Figure 2F, the Young’s moduli of the Gel-Ph/HA-Ph hydrogels were similar to each other and significantly higher than that of the gelatin hydrogel, suggesting that the Gel-Ph/HA-Ph hydrogels had similarly high network densities. The crushing stress of the hydrogels represented the greatest compressive stress before the failure of the network (stress dropped) (Figure 2G). The corresponding results revealed that the Gel-Ph/HA-Ph hydrogels with an increased HA-Ph content showed higher crushing stress, which occurred at higher compressive strain (Figure 2G). These results indicated that the addition of HA-Ph could increase the mechanical properties of the Gel-Ph-based hydrogels by improving the flexibility of the hydrogels, but not changing the network density.

### 3.3. Cytocompatibility of Gel-Ph/HA-Ph Hydrogels

**Figure 3:**
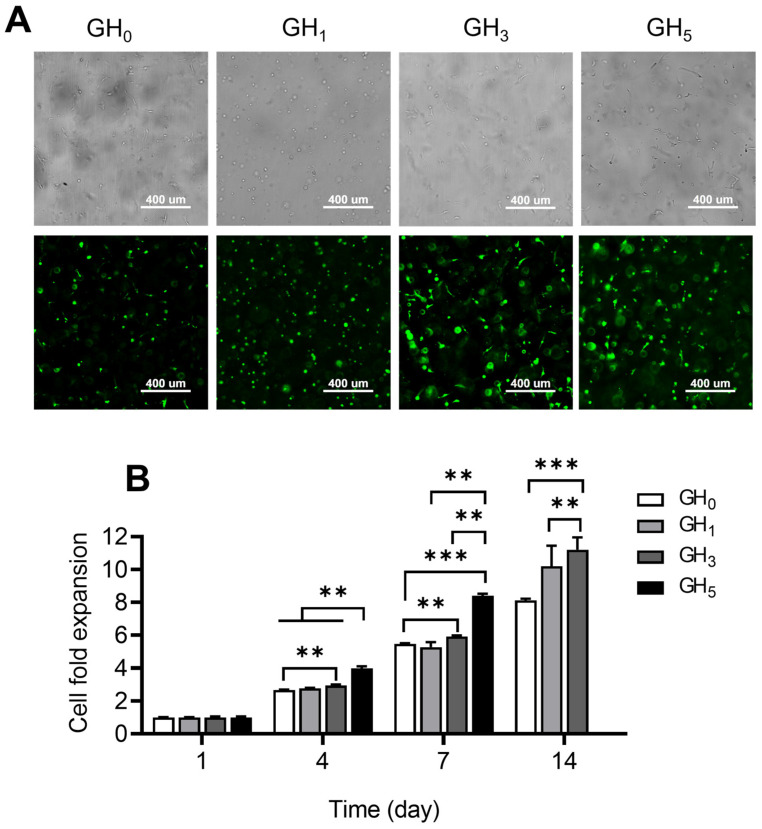
(**A**) Bright field images and live/dead-stained images of HBMSCs in Gel-Ph/HA-Ph hydrogels on day 5. Scale bars are 400 µm. (**B**) The time-dependent proliferation of HBMSCs within different Gel-Ph/HA-Ph hydrogels using CCK-8. ** and *** represent *p* < 0.01 and *p* < 0.001, respectively.

In order to evaluate the cytocompatibility of the Gel-Ph/HA-Ph hydrogels, the HBMSCs cultivated in different hydrogels were detected using CCK-8 assay and live/dead staining, respectively. As shown in Figure 3A, the HBMSCs in different Gel-Ph/HA-Ph hydrogels exhibited high viability with a negligible number of dead cells on day 5. For the cell proliferation results shown in Figure 3B, the viability of the HBMSCs in the Gel-Ph/HA-Ph hydrogels gradually increased up to 14 days, unless the GH_5_ and the cell proliferation were more obvious with the increase in the HA concentration. The result for GH_5_ on day 14 was unavailable because of the high-water content and collapse of the GH_5_ hydrogel (due to a high concentration of HA). Both Gel and HA are highly biocompatible materials. The current data showed that the crosslinked Gel-Ph/HA-Ph hydrogels also had excellent cytocompatibility where cell viability was >99% [37].

### 3.4. Surface-Guided Formation of Multicellular Spheroids and Further Encapsulation in Gel-Ph/HA-Ph Hydrogels

**Figure 4:**
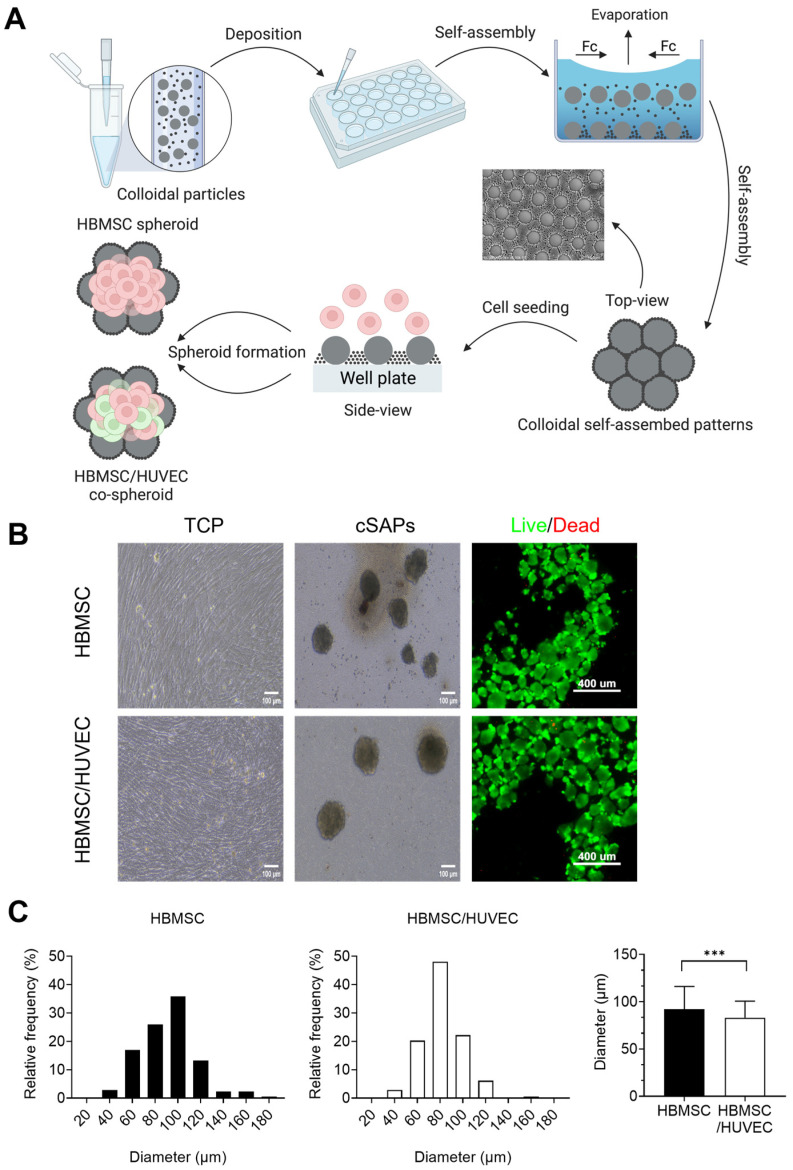
(**A**) Schematic diagram showing the preparation of cSAPs and the formation of multicellular spheroids on cSAPs; (**B**) morphology and live/dead staining of HBMSC spheroids and HBMSC/HUVEC co-spheroids on cSAPs after seeding for 48 h; scale bars are 100 and 400 µm; (**C**) proportional distribution of HBSC spheroids (**left**), HBMSC/HUVEC co-spheroids (**middle**) in different diameter ranges and statistical analysis of the diameters of multicellular spheroids (**right**). *** represent *p* < 0.001, respectively.

The preparation of the cSAPs and the formation of the multicellular spheroids are illustrated in Figure 4A. The SEM images showed the surface topography of the cSAPs layers, which was highly ordered, with 0.4 μm PS small particles surrounding the 5 μm Si large particles, forming a compact hexagonal structure, as previously reported [22,38,39,40,41]. Subsequently, the HBMSCs and HBMSCs/HUVECs were seeded onto the surface of the cSAPs at a high density to form multicellular spheroids within 48 h, as shown in Figure 4B. The statistical analysis demonstrated that the average diameter of the HBMSC spheroids and HBMSC/HUVEC co-spheroids was 92.1 ± 23.9 μm and 83.1 ± 17.5 μm (Figure 4C), respectively. In terms of the size distribution of the multicellular spheroids, the HBMSC/HUVEC co-spheroids were more uniform than the HBMSC spheroids. The size of the spheroids was decisive to the survival of cells in the spheroids, so a live/dead staining was performed to evaluate the cell viability. As shown in Figure 4B, from the inner core to the exterior, the spheroids displayed a high degree of viability with a negligible number of dead cells. It was well-recognized that spheroids larger than 200 μm could undergo necrosis due to the reduced diffusivity of oxygen and nutrients [17,42,43,44,45,46]. Therefore, the HBMSC spheroids and HBMSC/HUVEC co-spheroids prepared in our study could maintain excellent cell viability, as their sizes were less than 200 μm.

**Figure 5:**
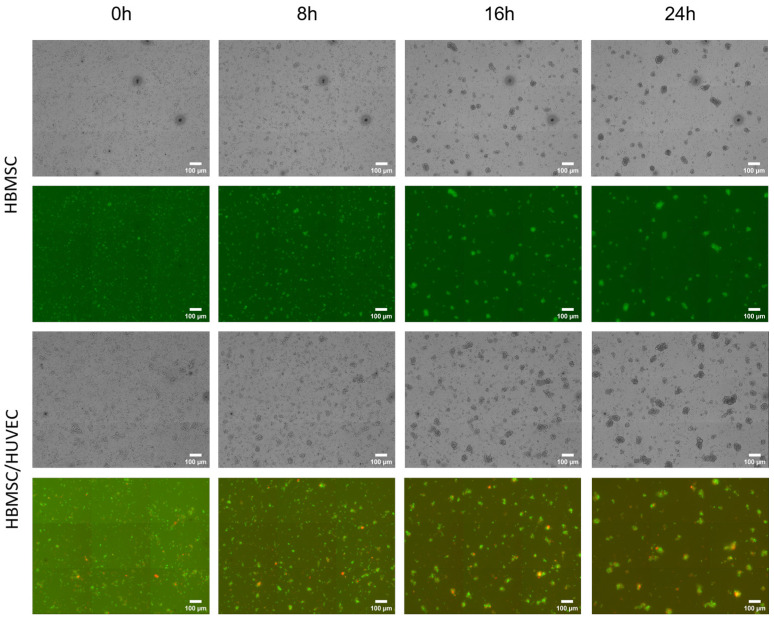
Monitoring the formation of HBMSC spheroids and HBMSC/HUVEC co-spheroids on cSAPs using the JuLI Stage Living cell monitoring system within 24 h after inoculation; the pictures at 0 h, 8 h, 16 h and 24 h show the dynamic formation process of multicellular spheroids. Scale bars are 100 µm.

To better understand how the HBMSCs and HBMSCs/HUVECs formed the multicellular spheroids on the surface of the cSAPs, we stained the HBMSCs and HUVECs with PKH67 and PKH26, respectively, and then monitored the process of HBMSC and HBMSC/HUVEC pelletization with a live-cell imaging system. The results in Figure 5 showed that both types of cells migrated rapidly on the surface of the cSAPs and then aggregated into multicellular spheroids.

**Figure 6:**
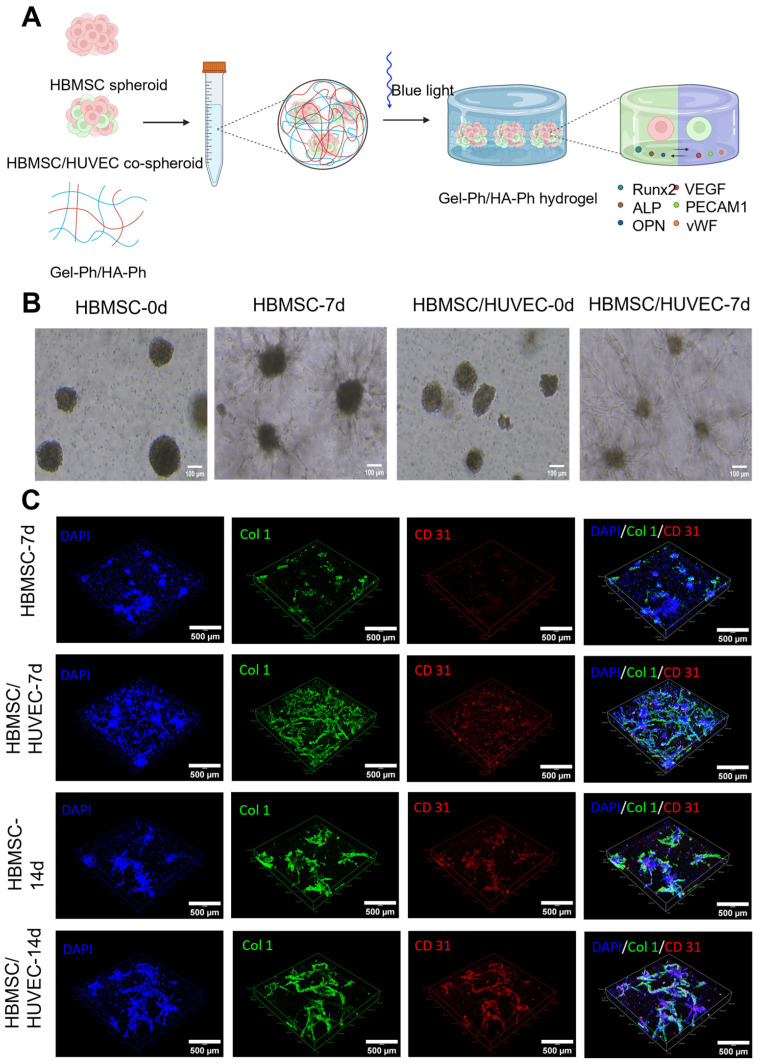
(**A**) Schematic diagram showing the encapsulation of HBMSC spheroids and HBMSC/HUVEC co-spheroids into Gel-Ph/HA-Ph hydrogels; (**B**) the morphology of HBMSC spheroids and HBMSC/HUVEC co-spheroids in Gel-Ph/HA-Ph hydrogels at day 0 and day 7; scale bars are 100 µm. (**C**) immunofluorescence staining of bone formation marker Col1α1 (green) and angiogenesis marker CD31 (red) after encapsulating and culturing the HBMSC spheroids and HBMSC/HUVEC co-spheroids in Gel-Ph/HA-Ph hydrogels for 7 and 14 days; scale bars are 500 μm.

In order to maintain the 3D culture of the multicellular spheroids, the HBMSC spheroids and HBMSC/HUVEC co-spheroids were further encapsulated in Gel-Ph/HA-Ph hydrogels (Figure 6A), respectively. The morphological features of the multicellular spheroids in the hydrogels at day 0 and day 7 are shown in Figure 6B, indicating a distinct branch outgrowth of cell spheroids over time. These spheroid branches were interconnected to form a dense network facilitating the connections among spheroids, which was highly consistent with previous reports [47,48,49,50,51]. In conclusion, the good biocompatibility and macroporous characteristics of the Gel-Ph/HA-Ph hydrogels were the main reasons for maintaining the long-term viability of cell spheroids, which was beneficial to the subsequent evaluation of the osteogenic and angiogenic capabilities of the cell spheroids.

### 3.5. Angiogenic/Osteogenic Differentiation of Multicellular Spheroids in Gel-Ph/HA-Ph Hydrogels In Vitro

In the next step, the angiogenic and osteogenic capabilities of the HBMSC spheroids and HBMSC/HUVEC co-spheroids in the Gel-Ph/HA-Ph hydrogels were evaluated by examining the expressions of CD31 and Col1a1 at the protein levels, respectively. As shown in Figure 6C, the cells in both groups showed a high expression of Col1a1 upon osteogenic induction, and the fluorescent intensity of Col1a1 increased significantly on day 14, compared with that on day 7. Interestingly, the HBMSC/HUVEC co-spheroids expressed a higher level of Col1a1 than the HBMSC spheroids at the same timepoint. The immunofluorescent staining images of CD31 showed that the HBMSC/HUVEC co-spheroids expressed a significantly higher level of CD31 than the HBMSC spheroids at day 7 and day 14. Most importantly, the cells in the HBMSC/HUVEC co-spheroids showed a better sprouting capability compared with those in the HBMSC spheroids, which was consistent with previous reports [52,53].

**Figure 7:**
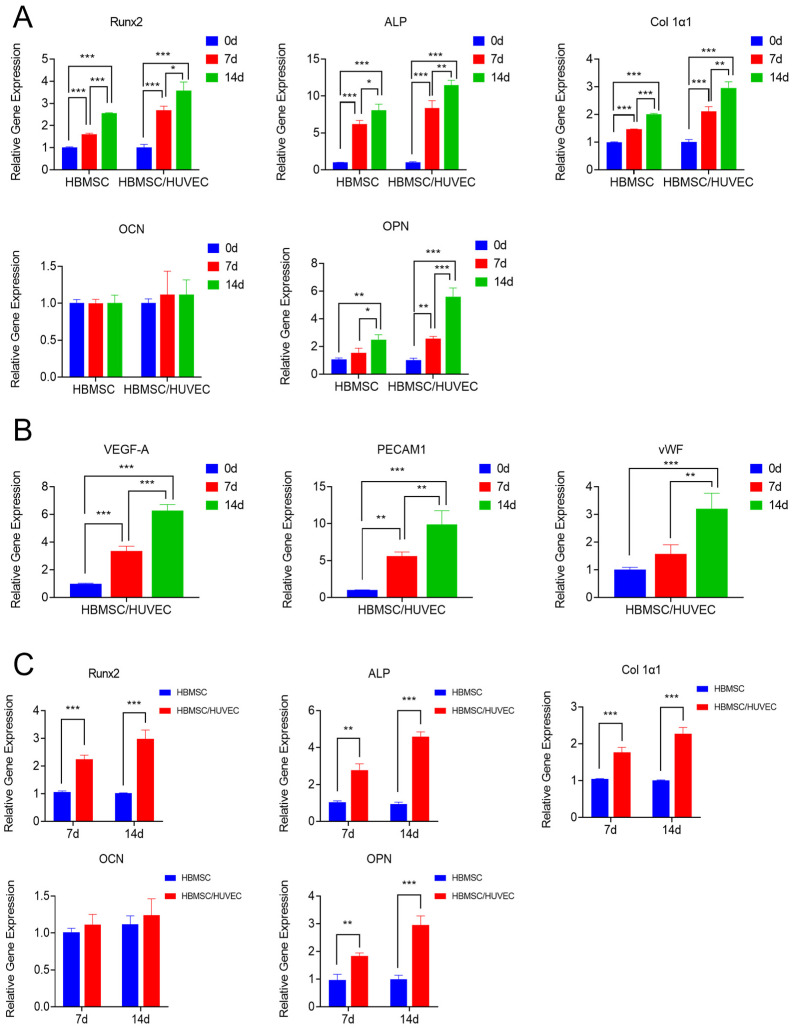
(**A**) Expressions of osteogenesis-related genes after encapsulating and culturing the HBMSC spheroids and HBMSC/HUVEC co-spheroids in Gel-Ph/HA-Ph hydrogels for 7 and 14 days; (**B**) expressions of angiogenesis-related genes after encapsulating and culturing the HBMSC spheroids and HBMSC/HUVEC co-spheroids in Gel-Ph/HA-Ph hydrogels for 7 and 14 days; (**C**) RT-qPCR detection of mRNA expression of osteogenic differentiation-related genes in HBMSC spheroids and HBMSC/HUVEC co-spheroids cultured for 7 and 14 days in Gel-Ph/HA-Ph hydrogels. *, ** and *** represent *p* < 0.05, *p* < 0.01 and *p* < 0.001, respectively.

Furthermore, the osteogenic and angiogenic gene expressions of the cell spheroids embedded in the Gel-Ph/HA-Ph hydrogels were examined at different timepoints. As shown in Figure 7A, the expression of the Runx2, ALP, Col1α1 and OPN genes in the HBMSC spheroids and HBMSC/HUVEC co-spheroids increased significantly at day 7 and day 14 compared to day 0. In the meantime, the expression of the VEGF-A, PECAM1 and vWF genes in the HBMSC/HUVEC co-spheroids at day 7 and day 14 was much higher than at day 0 (Figure 7B). Most importantly, the HBMSC/HUVEC co-spheroids showed a significantly higher expression of the Runx2, ALP, Col1α1 and OPN genes compared to the HBMSC spheroids over time (Figure 7A), which was in line with previous studies [52,53]. These results suggested that osteogenesis and angiogenesis could be effectively promoted through the 3D culture of HBMSC/HUVEC co-spheroids in Gel-Ph/HA-Ph hydrogels.

### 3.6. The Effects of Multicellular Spheroids in Gel-Ph/HA-Ph Hydrogels In Vivo

**Figure 8:**
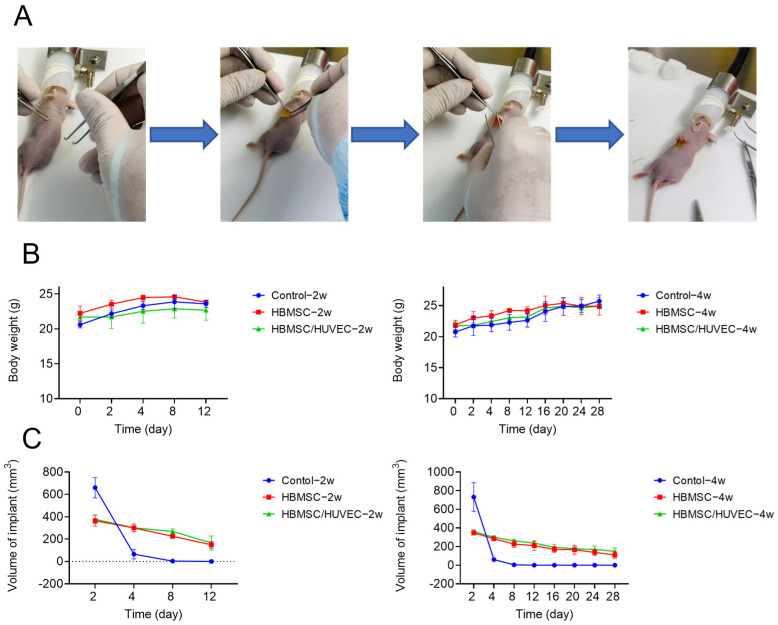
(**A**) Procedure of subcutaneous implantation of Gel-Ph/HA-Ph hydrogels into nude mice; (**B**) the body weight of different experimental mice after 2 and 4 weeks post-implantation; (**C**) the volume changes in different Gel-Ph/HA-Ph hydrogels at the implantation sites within 2 and 4 weeks.

To assess the vasculogenic and osteogenic effects of both multicellular spheroids in vivo, the hydrogels encapsulating the HBMSC spheroids and HBMSC/HUVEC co-spheroids were subcutaneously implanted into nude mice for 2 and 4 weeks (Figure 8A), and the hydrogel without cells was used as a blank control. It should be mentioned that the body weight of the nude mice in each group increased slowly and with negligible differences after 2 and 4 weeks of implantation (Figure 8B), which verified the biosafety of our strategy. Meanwhile, we measured the volume of the hydrogels at the implantation site and found that the volume of the hydrogels with cell spheroids (the HBMSC group and HBMSC/HUVEC group) decreased slowly and synchronously after implantation, whereas the hydrogel of the blank control group swelled rapidly during the initial 2 days, and then almost completely degraded and disappeared to unmeasurable levels on day 4 (Figure 8C).

**Figure 9:**
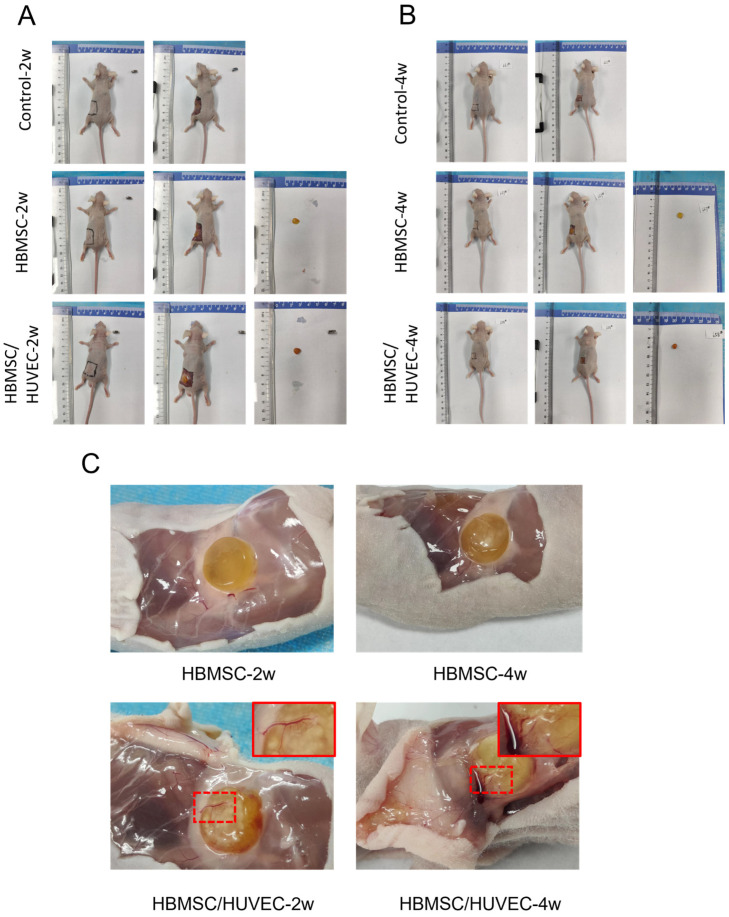
(**A**,**B**) The skin tissue and surrounding tissue isolated after 2 and 4 weeks post-implantation; (**C**) the blood vessel growth in the Gel-Ph/HA-Ph hydrogels at the implantation sites after 2 and 4 weeks post-implantation; the blood vessels are marked with the red rectangular border.

The experimental mice were sacrificed after 2 and 4 weeks of implantation (Figure 9A,B), and the status of the hydrogel at the implantation site was observed in each group. It was found that the neovascularization of the HBMSC/HUVEC group was more pronounced than that of the HBMSC group at both timepoints (Figure 9C).

**Figure 10:**
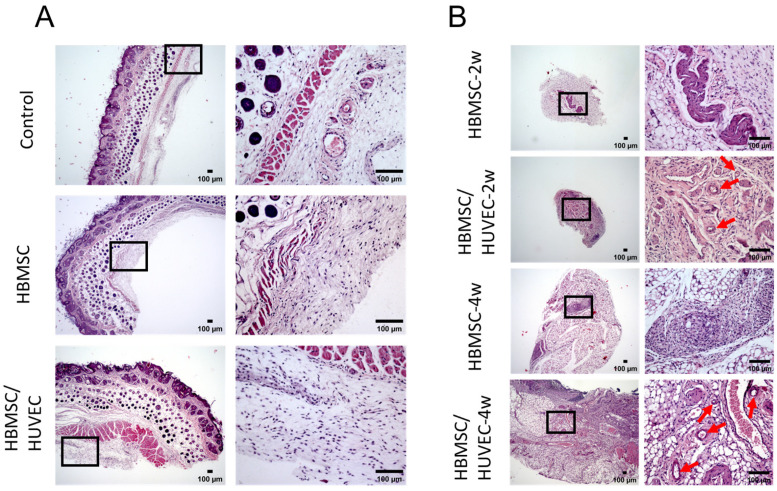
(**A**) H&E staining of the skin tissues from implantation sites after 4 weeks post-implantation; scale bars are 100 μm. (**B**) H&E staining of the Gel-Ph/HA-Ph hydrogels encapsulating HBMSC spheroids (HBMSC) and HBMSC/HUVEC co-spheroids (HBMSC/HUVEC) after 2 and 4 weeks (2w: 2 weeks; 4w: 4 weeks) post-implantation; the circles pointed out by the red arrows are the structures of the blood vessels; scale bars are 100 μm.

The harvested samples in different groups were further subjected to histopathological staining. As shown in Figure 10A, the immune response of the surrounding skin tissue was relatively limited, as the boundary between the implant and the dermis was clear and no damage was found in the surrounding tissue (Figure 10A). More importantly, the vascular structure detected in the HBMSC/HUVEC group was more significant than that of the HBMSC group (Figure 10B).

**Figure 11:**
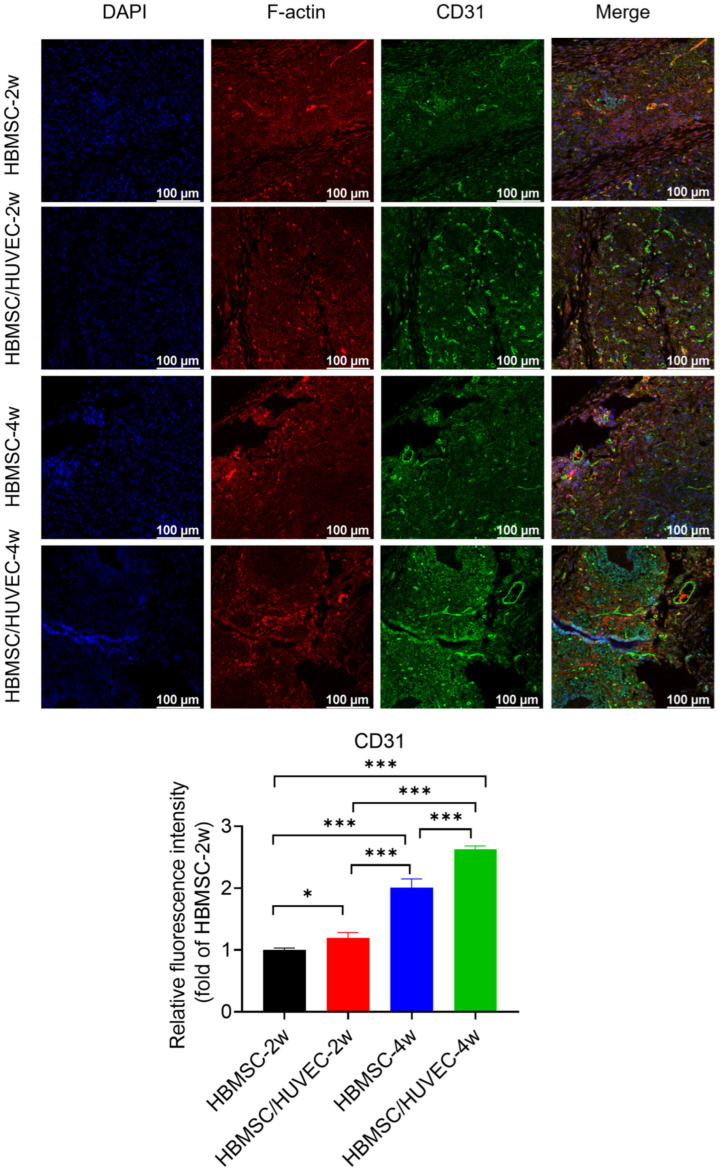
Immunofluorescent staining of the Gel-Ph/HA-Ph hydrogels encapsulating HBMSC spheroids (HBMSC) and HBMSC/HUVEC co-spheroids (HBMSC/HUVEC) with angiogenic marker CD31 (green) after 2 and 4 weeks (2w: 2 weeks; 4w: 4 weeks) post-implantation. The nuclei and F-actin were co-stained with DAPI (blue) and phalloidin (red), respectively. Scale bars are 100 μm. The fluorescence intensity of CD31 was semi-quantified (*n* = 5). * and *** represent *p* < 0.05 and *p* < 0.001, respectively.

Furthermore, as the results of the immunofluorescent staining show in Figure 11, the HBMSC/HUVEC group exhibited a higher expression of the vascular marker CD31 than the HBMSC group in vivo, which was consistent with the neovascularization result shown in Figure 9C.

## 4. Discussion

The generation of multicellular spheroids from a nanopatterned substrate is a novel way of studying cell–cell interactions, which can be used to simulate tissue development in vitro. The format of multicellular spheroids can better reflect the complexity and heterogeneity of native tissues, strengthening the interaction between cells to better mimic cell behaviors in vivo [54]. In addition, the production of growth factors, cytokines and ECM within the cell spheroids can also affect cell behaviors [55,56]. In previous studies, various coculture systems of mesenchymal stem cells (MSCs) and endothelial cells (ECs) were developed to promote osteogenesis and angiogenesis [57,58,59,60]. For example, ECs can promote the osteogenic differentiation of MSCs [61,62,63] and further mineralization [63], and MSCs can promote the sprouting of ECs and induce the formation of a prevascular network [64,65,66,67]. In cocultures, paracrine signaling between MSCs and ECs also plays a key role. On the one hand, MSCs can enhance the viability and vascularization of ECs by secreting VEGF [68,69]. On the other hand, ECs can promote the proliferation and differentiation of MSCs by secreting BMP-2 [57,59]. An increasing number of studies have shown that, compared with indirect coculture methods, such as the utilization of conditioned media or transwell inserts, the direct cell–cell contact of MSCs and ECs is closer to the actual conditions [57,70]. In view of this, we believe that multicellular spheroids with improved interactions between heterogenic cells are preferable. In the present study, we demonstrated that the in vitro cultivation of HBMSC/HUVEC co-spheroids in Gel-Ph/HA-Ph hydrogels could upregulate the expression of osteogenic genes, including Runx2, ALP, Col 1α1 and OPN, as well as the expression of angiogenic genes such as VEGF-A, PECAM1 and vWF, on day 7 and day 14. The subcutaneous implantation further verified that the hydrogels encapsulating the HBMSC/HUVEC co-spheroids were favorable to angiogenesis. The formation and maturation of blood vessels in the hydrogels may be attributed to the specific proangiogenic cytokines secreted by the HBMSCs [68,69]. However, unlike the results of the in vitro culture, the HBMSC/HUVEC co-spheroids in our study did not exhibit signs of facilitating osteogenesis in vivo. No effective bone matrix or mineralized bone tissue appeared in the hydrogels encapsulating the cell spheroids within 4 weeks (data not shown). It was considered that the subcutaneous environment of the nude mice was absent of exogenous osteoinductive factors, which may not be conducive to the osteogenic differentiation of HBMSCs. Instead, the undifferentiated HBMSCs may facilitate the vascular differentiation of HUVECs.

Previously, we evaluated cell behaviors, such as the adhesion and differentiation of other stem cells, on the surface of cSAPs [22,38,39,71]. In this study, the cSAPs were used as a cell culture platform, and it was found that HBMSCs at a low-density (2 × 10^4^ cells/mL) could spread (data not shown) on top, whereas, at a high density (1 × 10^5^ cells/mL), they aggregated into multicellular spheroids. In addition, the size of the multicellular spheroids could be flexibly adjusted via cell the seeding density and culture time. Here, we optimized the cell density and culture time to 1 × 10^5^ cells/mL and 48 h, respectively. The obtained HBMSC spheroids and HBMSC/HUVEC co-spheroids were of a size below 100 μm, with good uniformity. The size of the spheroids was decisive to the survival state of the cells inside them. Previous studies have demonstrated that a size of spheroids below 200 μm did not affect the transport of oxygen and nutrients inside the spheroids [17,42,43,44,45,46]. In addition, multicellular spheroids formed through spontaneous self-assembly on the surface of cSAPs were more conducive to the survival of multicellular spheroids than cell aggregation formed with the spinner flask method or other external forces. Therefore, the multicellular spheroids prepared in our study exhibited excellent cell viability, which enabled the subsequent evaluation of osteogenic and angiogenic performances. More importantly, compared to multicellular spheroids prepared through other methods, such as the fabrication of hydrogel scaffolds [72,73], the multicellular spheroids formed on cSAPs were more convenient for collection and further single-factor biological analyses. The increase in surface roughness could inhibit the adsorption of cell adhesion-related proteins on biomaterials, thereby minimizing the cell–matrix interaction and promoting the cell–cell interaction [11]. Therefore, we believe that the surface roughness of the cSAP samples was the main factor that caused cells to aggregate into spheroids.

cSAPs, as a platform for multicellular spheroids, provided the advantages of high efficiency for spheroid formation and easy collection. However, since the multicellular spheroids were in a semi-suspended state, there was a certain loss of spheroids when changing the medium. Therefore, in order to maintain cell viability, 3D cultures are the first choice for cultivating multicellular spheroids. Here, we used blue-light cross-linked Gel-Ph/HA-Ph hydrogels to encapsulate HBMSC spheroids and HBMSC/HUVEC co-spheroids, respectively. The high cell viability of the HBMSC in different Gel-Ph/HA-Ph hydrogels demonstrated that all hydrogels had excellent biosafety and biocompatibility. The enhanced cell proliferation level with the increase in the HA concentration indicated the great advantage of using HA for stem cell expansion, and the high porosity in the GH_5_ hydrogels might have contributed to the cell proliferation inside. The employment of Gel-Ph/HA-Ph hydrogels provided the following four main advantages: First, the gelatin and HA are natural biopolymers, and their hybrid hydrogels can mimic the extracellular matrix structure with excellent biocompatibility and biodegradability [74,75]. Second, it has been reported that gelatin is a component that can promote cell adhesion and proliferation [76,77,78], and HA has been widely used to maintain the differentiation potential of stem cells [79,80] and promote angiogenesis [81]; therefore, hybrid hydrogel scaffolds combining gelatin and HA have a wide range of applications in tissue engineering [31,35,75,81,82]. Third, it is well known that common UV-crosslinked hydrogels may induce potential risks of chromosomal and genetic instability [83], and Gel-Ph/HA-Ph hydrogels prepared by using the blue-light crosslinking system can avoid these problems. Last, but not least, the change in the HA-Ph content did not significantly affect the elastic modulus of the prepared Gel-Ph/HA-Ph hydrogels, but the internal pores of the Gel-Ph/HA-Ph hydrogels could be tuned with the increase in the HA-Ph content. This was due to the high-water absorption and retention capacity of HA-Ph, which led to the formation of larger pores in the hydrogels after drying. It was reported that the presence of the amide and carboxylic acid groups in the repeating unit of HA facilitates the water absorption ability [84]. The macroporous feature of the hydrogels is conducive to the encapsulation of multicellular spheroids and nutrient exchange, thereby being favorable to the biological activity of multicellular spheroids. In this study, we observed that cell behavior was related to the physicochemical property of the hydrogels and cell–cell interactions. Although the pore size is one of the important properties for cell migration and outgrowth, the mechanical and biochemical properties of the hydrogels are also critical. In the early culture timepoints (<7 days), the HBMSCs appeared to have an elongated morphology in the GH_3_ and GH_5_ hydrogels, which may be attributed to the higher content of HA or the large pore sizes.

In the present study, it was interesting to note that the subcutaneously implanted Gel-Ph/HA-Ph hydrogels without cells degraded rapidly within 4 days, whereas the Gel-Ph/HA-Ph hydrogels with multicellular spheroids could be maintained until the end of the in vivo experiment (4 weeks). It is possible that the multicellular spheroids in the hydrogels could secrete a large amount of an extracellular matrix to increase the strength of the internal network, thereby delaying the degradation of the hydrogels in vivo. We could conclude from the high expression of type one collagen genes and proteins described above that the multicellular spheroids facilitated the secretion of extracellular matrix base-associated proteins, which was consistent with the conclusions of previous reports [11,85]. The H&E staining results further demonstrated that the Gel-Ph/HA-Ph hydrogels were biocompatible and induced a limited immune response. These results demonstrated that the use of Gel-Ph/HA-Ph hydrogels encapsulating HBMSC/HUVEC co-spheroids is a new form of studying cell–cell interactions, which also provides a meaningful theoretical basis for the construction of organoids.

In this study, we prepared the HBMSC spheroids and HBMSC/HUVEC co-spheroids, respectively, and then encapsulated them in Gel-Ph/HA-Ph hydrogels to evaluate their osteogenic and angiogenic performances. Compared to the HBMSC spheroids, the HBMSC/HUVEC co-spheroids showed upregulated levels of osteogenic and angiogenic genes. More importantly, the in vivo experiments demonstrated that the HBMSC/HUVEC co-spheroids could significantly promote the angiogenesis of nude mice after a subcutaneous implantation, which is a key factor to ensuring nutrient transport in bone tissue engineering. These results demonstrated that the HBMSC/HUVEC co-spheroids with promoted cell–cell communication could enhance osteogenesis and angiogenesis for bone regeneration.

## 5. Conclusions

Hybrid multicellular spheroids were fabricated using a nanopatterned substrate, named cSAPs, for the first time to the best of our knowledge. Both HBMSCs and HUVECs could attach to the cSAP substrate, and then gradually formed multicellular spheroids via cell migration and proliferation. The nanopattern-derived formation of multicellular spheroids showed numerous benefits, such as self-organization, size uniformity and easy collection. The phenol-modified hydrogels could be crosslinked via blue light with high cyto-biocompatibility. The optimized hydrogel, GH_3_, was used to encapsulate the cell spheroids. The hybrid HBMSC/HUVEC co-spheroids expressed higher levels of osteogenic and angiogenic genes than the HBMSC spheroids, indicating that the cell–cell interaction benefited osteogenesis and angiogenesis. In vivo, the HBMSC/HUVEC co-spheroids showed a better proangiogenic effect than the HBMSC spheroids. This study developed a biotechnology for multicellular spheroids, which could be used for organoid generation. This study also provided new insights into cell–cell interactions for tissue regeneration.

## Figures and Tables

**Table 1:** The primer sequences used for RT-qPCR analysis.

Gene Name	Primer Sequences (5′ → 3′)
GAPDH	F: TCGGAGTCAACGGATTTGGT
	R: TTCCCGTTCTCAGCCTTGAC
Runx2	F: CCGCCTCAGTGATTTAGGGC
	R: GGGTCTGTAATCTGACTCTGTCC
ALP	F: AACATCAGGGACATTGACGTG
	R: GTATCTCGGTTTGAAGCTCTTCC
Col lα1	F: GTCACCCACCGACCAAGAAACC
	R: AAGTCCAGGCTGTCCAGGGATG
OCN	F: CTCACACTCCTCGCCCTATT
	R: TTGATACAGGTAGCGCCTGG
OPN	F: GCCGAGGTGATAGTGTGGTT
	R: AACGGGGATGGCCTTGTATG
vWF	F: AGAACAGATGTGTGGCCCTG
	R: CTTCCGGTCCTGACAGACAC
PECAM1	F: CCAAGGTGGGATCGTGAGG
	R: TCGGAAGGATAAAACGCGGTC
VEGF-A	F: GGCCTCCGAAACCATGAACT
	R: GGTCTCGATTGGATGGCAGT

## Data Availability

Not applicable.
